# A Mid-Infrared Perfect Metasurface Absorber with Tri-Band Broadband Scalability

**DOI:** 10.3390/nano14151316

**Published:** 2024-08-05

**Authors:** Yongtu Zou, Shaolin Zhou, Jingxi Li, Shanri Chen, Zhijian Chen

**Affiliations:** The School of Microelectronics, South China University of Technology, Guangzhou 511442, China; 202121061703@mail.scut.edu.cn (Y.Z.); eeslzhou@scut.edu.cn (S.Z.); 202220160942@mail.scut.edu.cn (J.L.); 202210192705@mail.scut.edu.cn (S.C.)

**Keywords:** metasurface absorber, tri-broadband, infrared atmospheric windows, multipole resonance

## Abstract

Metasurfaces have emerged as a unique group of two-dimensional ultra-compact subwavelength devices for perfect wave absorption due to their exceptional capabilities of light modulation. Nonetheless, achieving high absorption, particularly with multi-band broadband scalability for specialized scenarios, remains a challenge. As an example, the presence of atmospheric windows, as dictated by special gas molecules in different infrared regions, highly demands such scalable modulation abilities for multi-band absorption and filtration. Herein, by leveraging the hybrid effect of Fabry–Perot resonance, magnetic dipole resonance and electric dipole resonance, we achieved multi-broadband absorptivity in three prominent infrared atmospheric windows concurrently, with an average absorptivity of 87.6% in the short-wave infrared region (1.4–1.7 μm), 92.7% in the mid-wave infrared region (3.2–5 μm) and 92.4% in the long-wave infrared region (8–13 μm), respectively. The well-confirmed absorption spectra along with its adaptation to varied incident angles and polarization angles of radiations reveal great potential for fields like infrared imaging, photodetection and communication.

## 1. Introduction

In recent decades, metasurface absorbers (MSAs) have emerged as ultra-compact two-dimensional (2D) devices for ideal wave absorption. With significant attention paid to MSAs, typical architectures such as periodic metal–insulator–metal (MIM) MSAs [1], all metal MSAs [2], all-dielectric MSAs [3], hybrid MSAs [4] and even disordered MSAs [5,6] have been proposed, which exhibit great absorptivity performance for potential applications. Fundamentally, the essential principle of ideal wave absorption design is to probe the condition of impedance matching. However, the required calculation of the scattering-parameter-related lumped characteristic impedance is always cumbersome [7,8], thus limiting the practical implementations of impedance matching. At present, the primary method of MSA design is to excite different resonance modes by using various types of nanoresonators [9,10]. Therefore, besides the applications of stealth technologies [11] and thermal dissipation management [12], the intense local electric field enhancement by localized resonance can be employed in applications such as lasing [13], hot-electron engineering [14] and detection enhancement [15]. Furthermore, when considering many practical scenarios such as photodetectors [16], bolometers [17], energy harvesting [18] and stealth technologies [19], the absorption bandwidth is an essential factor that is directly related to other figures of merits such as the responsivity, efficiency and so on, since multiple wavelength ranges are usually involved instead of a single one. Therefore, there has been considerable attention and intensive investigations focused on broadband MSAs in recent years [20].

An intuitive method to expand the absorption bandwidth involves coupling adjacent resonance absorption peaks induced by nanoresonators with similar sizes and shapes arranged either perpendicular [21,22] or parallel [23,24] to the incident wave. However, the relevant costs to fabricate such multi-level composite structures can be substantial. Instead, broadband absorption can also be achieved by coupling multiple resonance modes excited by one single type of structure, such as nichrome crosses [25], ITO grids [26] and titanium blocks [27]. However, excitation of multiple resonances is not always straightforward, leading to a delicate complexity trade-off between the process of fabrication and structural design. On the other hand, by individually taking multiple resonance peaks into different wavelength ranges, i.e., the so-called multi-band modulation [22,28,29], narrow band absorption tends to be generated due to the limited quantity of resonance peaks.

In recent years, myriads of studies on multi-band broadband modulation in infrared atmospheric windows have emerged for infrared camouflage [30,31]. For the sake of low emissivity, devices are usually required to exhibit minimal absorption in multiple atmospheric windows, according to the Kirchhoff Law. Other related works like absorption modulations [32] and active switches [33] in both the mid-wave infrared (MWIR, 3–5 μm) and the long-wave infrared (LWIR, 8–14 μm) atmospheric windows also highlight the potential of multi-broadband absorption modulations. However, to the best of our knowledge, only a few dual-broadband metasurface absorbers have been reported to operate effectively across both the MWIR and the LWIR regions [27,34]. Nonetheless, the bandwidth and absorption efficiency are limited and far from ideal. Further, due to the size constraints, concurrently achieving absorption in the short-wave infrared (SWIR), MWIR and LWIR regions remains a challenge.

In this paper, we propose a scheme for a tri-band broadband MSA that targets the infrared atmospheric windows. In the SWIR (1.4–1.7 μm) region, the average absorption reaches 87.6% due to a high order Fabry–Perot (F-P) resonance mode. In the MWIR (3.2–5 μm) region, an average absorption of 93.2% is achieved by resorting to the low-order FP resonance, the magnetic dipole (MD) resonance and the electric dipole (ED) resonance. In the LWIR (8–13 μm) region, an average power absorption of 92.6% is obtained by coupling four independent ED resonance modes across different wavelengths. Each resonance mode is deliberately leveraged and adjusted in as-required wavelength regions though the numerically calculated electric field profiles and optimized process of parametric sweeping. Finally, our scheme prominently features the excellent performance of multi-band absorption with its large bandwidth, high absorption efficiency and relative simplicity of structure design as well as its insensitivity to the variations of polarization angle and incident angle, which well confirms the feasibility and adaptability of our scheme for potential applications in imaging, detection and communication.

## 2. Scheme and Analysis

### 2.1. Quality Factor of Nanoresonators

Essentially, to realize broadband absorption though couplings of multiple resonance modes, two preconditions must be satisfied: multiple resonance modes coexisting concurrently and a low quality (Q) factor. High-Q resonances tend to exhibit absorption peaks that are hardly intercoupled, causing an absorption spectrum with significant variance [35] compared with their low-Q counterparts [25]. Intrinsically, the Q factor relates to the loss and leakage rates within the resonance system. In general, it can be defined as $Q=\omega_{0}\frac{W}{P_{\mathrm{Loss}}}$, where $\omega_{0}$ is the angular frequency of incident light, W denotes the stored energy and $P_{\mathrm{Loss}}$ denotes energy loss due to scattering and resistive effects [36]. In the design of MSAs, the scattering power should be particularly low. As a result, as the resistive loss mainly results from the ohmic loss, the material property of as-selected meta-units or nanoresonators for constructing the MSA is a crucial factor.

**Figure 1:**
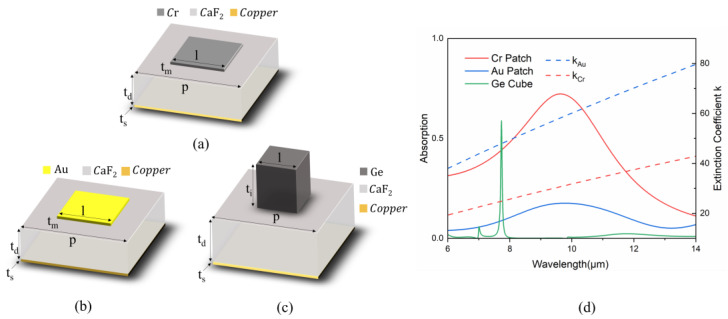
Schematic of metasurface absorbers constructed using three different types of nanoresonators: (**a**) a Cr patch, with period of p = 1.8 μm, length of patch l = 1.6 μm, thickness of t_m_ = 200 nm, on a dielectric spacer with thickness t_d_ = 600 nm and a bottom metal substrate thickness of t_s_ = 100 nm; (**b**) a Au patch with the same as that in the (**a**); (**c**) Ge Cube, with a period of 2.8 μm and length and thicknesses of *l* = 2.1 μm, t_i_ = 2.1 μm, t_d_ = 2.4 μm, t_s_ = 100 nm. (**d**) Calculated infrared absorption spectra (left axis) of the MSA with a Cr patch, Au patch and Ge cube. The extinction coefficient of Au and Cr are cited from [38,39] (right axis). Intrinsic Ge is lossless with a negligible extinction coefficient [40].

As illustrated in Figure 1a–c, three different MSAs were constructed according to the resonator–insulator–metal architecture customized with distinctly different nanoresonators, i.e., the Cr nanopatches, Au nanopatches and Ge nanocubes. As shown in Figure 1d for the absorption spectra, the dielectric MSA (green line) clearly exhibits the largest Q factor at the resonance wavelength due to its lossless dielectric nature, which indicates the limitation of such simple dielectric nanoresonators as broadband absorbers. However, higher conductivity is not always advantageous for MSA design because the skin depth (σ=λ2πIm(n)) is inversely proportional to the imaginary part of the refractive index [37]. Thus, electromagnetic waves cannot penetrate deeply into novel metals like gold, resulting in significant reflection and limited absorption. As depicted in Figure 1d, despite a higher imaginary part of the refractive index due to its higher conductivity, an MSA composed of Au patches demonstrates the lowest absorption (blue line) due to the largest reflective loss, which indicates that the novel metal is not an ideal candidate for constructing broadband metasurface absorbers. However, the metal Cr has a moderate complex refractive index between the lossless dielectric and the novel metal. As a result, the Cr-based MSA assumes a moderately low-Q resonance mode for broadband and high absorption, indicating that Cr could be an ideal candidate resonant material for the construction of MSAs.

### 2.2. Multiple Resonance Excitations

**Figure 2:**
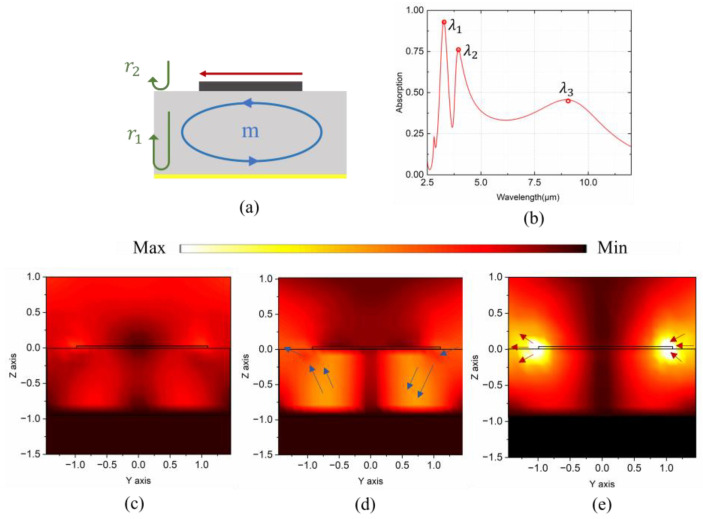
(**a**) The schematic of multi-mode coupling for broadband modulations of multi-band absorption. (**b**) The absorption spectrum of the MSA in Figure 1 with parameters of *p* = 3 μm, *l* = 2 μm, *t_m_* = 300 nm, t_d_ = 0.9 μm, and *t_s_* = 100 nm and electric intensity profiles in the YOZ plane at wavelengths of (**c**) λ_1_ = 3.26 μm, (**d**) λ_2_ = 3.95 μm, and (**e**) λ_3_ = 8.97 μm.

Based on the analyses above, multiple absorption peaks can be intentionally leveraged and coupled to generate the required multi-broadband spectra by resorting to various resonance mechanisms. As illustrated in Figure 2a, an FP resonance mode can be readily generated by the destructive interference of reflected waves from the top and bottom interfaces [37]. Additionally, it is known that metal strips aligned perpendicular to the direction of the electric field in a typical MIM architecture can induce an electric dipole resonance mode [41], and magnetic dipole (MD) resonance modes can be induced due to the reverse currents induced in the top and bottom metal layers above and below the middle dielectric spacer layer [42]. As indicated in Figure 1, Cr is selected for constructing the MIM-based MSA for optimized multi-broadband absorption.

As shown in Figure 2b, by deliberately adjusting the geometries of the Cr patch (Figure 1a) in the MIM-based MSA architecture, three distinct absorption peaks (λ_1_ to λ_3_) can be distinctly extracted and distinguished in the mid-infrared region. To reveal the mechanisms behind those resonant peaks, the extracted electric intensity distributions in the YOZ plane for the three peaks are also presented in Figure 2c–e. As denoted in Figure 2b and shown in Figure 2c–e, the electric field distribution at λ_1_ is obviously uniform and less fluctuant compared with that at λ_2_ and λ_3_. In Figure 2d, the electric field mainly concentrates inside the dielectric layer, where the electric vector (indicated by blue arrows) forms a half-ring with adjacent units. In contrast, Figure 2e exhibits the most intense localized electric field enhancement at the edge of the Cr patch, with the electric vector pointing from one side to the other (see red arrows). As confirmed by our previous work [43], the results in Figure 2c–e distinctly indicate the characteristics of electric field profiles corresponding to the FP mode, MD mode and ED mode, respectively.

As a result, such MIM-based MSAs constructed with a Cr-patch nanoresonator can effectively excite at least three distinct resonance modes for as-required broadband modulation of the absorptive spectrum. Considering the intrinsic property of the low Q factor of the Cr nanoresonator, it is reasonable and highly promising to achieve as-required multi-band modulation of broadband absorption spectrum by coupling the multiple modes with such resonant absorption peaks.

## 3. Results and Discussion

### 3.1. Model and Absorption Optimization

**Figure 3:**
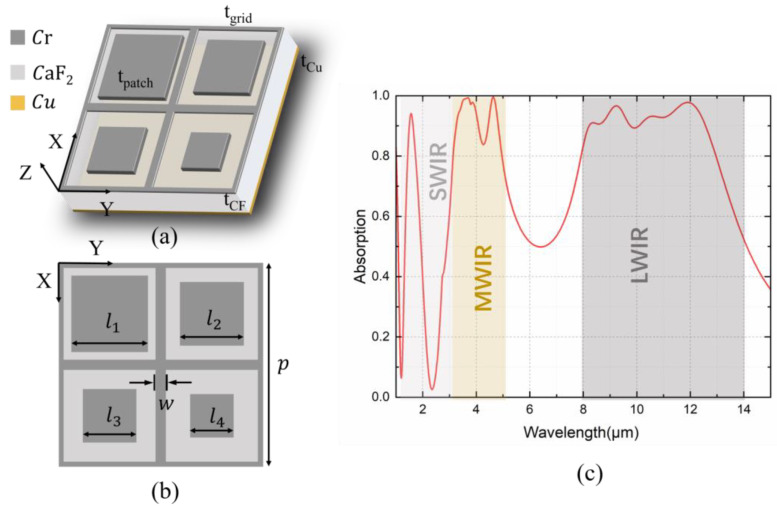
The schematic of the proposed MSA: (**a**) oblique view; (**b**) vertical view; (**c**) the absorption spectrum in the infrared region.

To further confirm the mechanism of multi-mode coupling for broadband modulation, the mid-infrared absorption spectrum is numerically calculated by the finite element method (FEM) using the commercial tool CST. A TE plane wave is incident with its electric field along the Y direction. The lateral boundary conditions of our MIM-based MSA are set as periodic boundaries. Based on the idea of coupling multi-mode resonant peaks for broadband absorption, the ultimate meta-unit of our MSA is optimally selected, as shown in Figure 3a,b. Specifically, the nanoresonator in the top layer consists of Cr grids and four Cr patches of varying sizes positioned in a four-quadrant manner.

The thickness of the grid and patches are *t_grid_ =* 5 nm and *t_patch_ =* 15 nm, respectively. The material comprising the dielectric layer is CaF_2_, with a thickness of *t_CF_ =* 0.9 μm, and a 100 nm metal copper layer is chosen as the substrate. The width of the Cr grid *w* is 0.4 μm, and the lengths of the patches are customized as *l*_1_ = 3 μm, *l*_2_ = 2.5 μm, *l*_3_ = 2.1 μm and *l*_4_ = 1.7 μm. The period of a unit cell is *p* = 8 μm. The refractive indices of Cr and CaF_2_ are referenced from [39,44]. The complex permittivity of copper is cited from the material library.

The absorption spectra are extracted as A = 1 − R, where R is the reflectivity given the negligible transmittance due to the almost fully reflective copper substrate. As shown in Figure 3c, the absorption spectrum optimally covers the entire infrared atmospheric window with distinctly enhanced multi-band broadband absorption. In the SWIR region, a low-Q resonance mode can be achieved at 1.83 μm with a power absorption approaching 88% and an average absorption of 87.6% from 1.4 μm to 1.7 μm. In the MWIR region, two major resonances are induced at 3.68 μm (A = 99.3%) and 4.64 μm (A = 99.6%). In a wavelength range (3.2–5 μm) nearly covering the entire MWIR, the average power absorption is up to 92.7%. Furthermore, our scheme realizes four absorption peaks in the LWIR region: 8.39 μm, 9.22 μm, 10.61 μm and 11.94 μm, with absorptions of 91%, 96.9%, 93.3% and 97.7%, respectively. In most of the LWIR regions (8–13 μm), the average absorption reaches 92.4%.

Finally, by leveraging seven resonant absorption modes, our scheme achieves broadband perfect absorption within three infrared atmospheric windows, with an average absorption of around 90%. As summarized in Table 1, our work demonstrates distinct advantages over other MSAs working in a similar infrared band in terms of the absorption bandwidth, efficiency and multi-band tunability.

### 3.2. Resonance Mode Tuning

**Figure 4:**
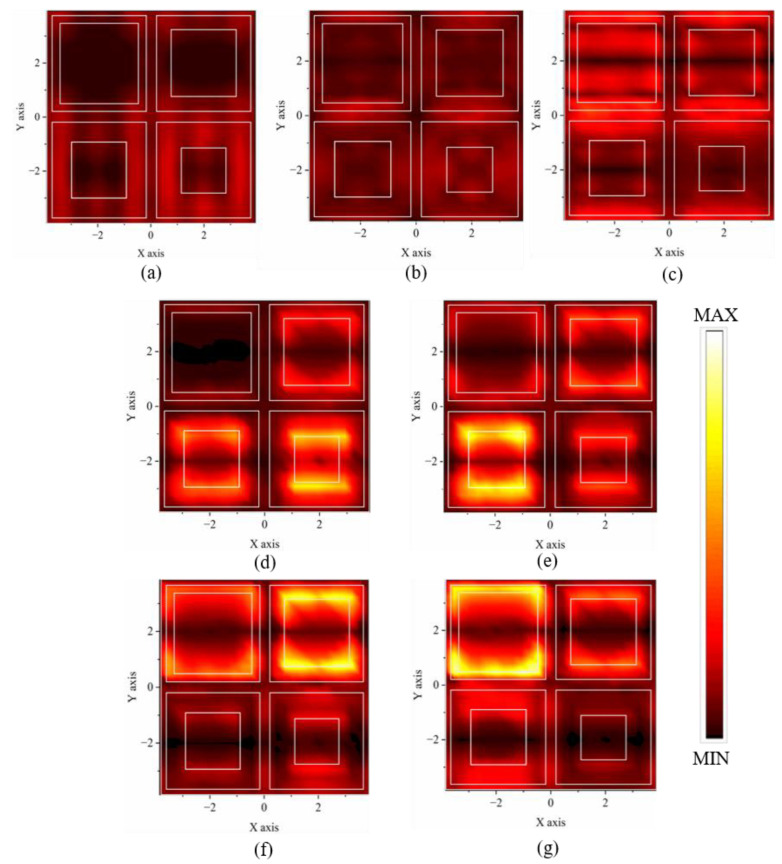
The electric field intensity profiles extracted in the XOY plane at seven resonance peaks at (**a**) λ_1_ = 1.57 μm, (**b**) λ_2_ = 3.68 μm, (**c**) λ_3_ = 4.64 μm, (**d**) λ_4_ = 8.39 μm, (**e**) λ_5_ = 9.22 μm, (**f**) λ_6_ = 10.61 μm and (**g**) λ_7_ = 11.94 μm. All electric field intensity profiles use the same reference scale.

**Figure A1:**
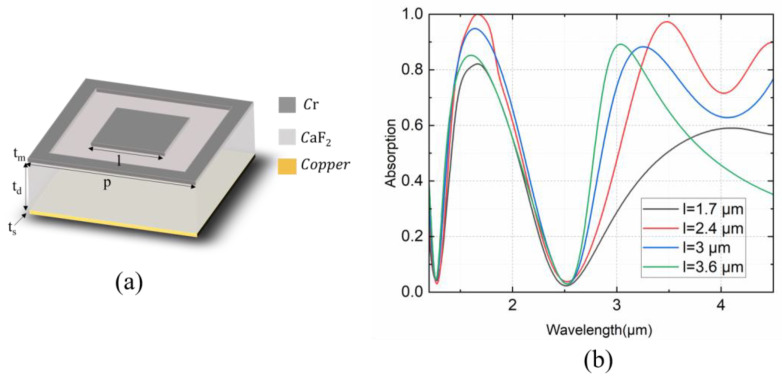
(**a**) The schematic of a single patch–grid structure. The structural parameters are as follows: p = 4 μm, l = 1.7 μm, t_m_ = 300 nm, t_d_ = 0.9 μm, t_m_ = 150 nm, w = 0.4 μm. (**b**) The absorption spectrum with different patch lengths.

**Figure A2:**
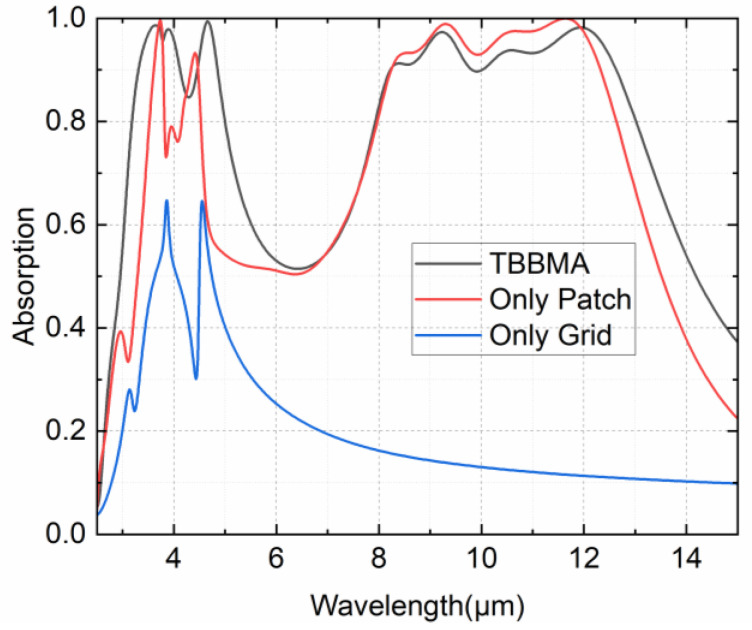
The absorption spectra of three types of MSA configured by different nanoresonators in the top layer. The setup using only the grid or only the patch shares the same parameters with our tri-band broadband MSA.

To verify the feasibility of our MSA scheme as proposed in Section 2, the electric field intensity profiles in the XOY plane are also extracted. In the ranges of SWIR and MWIR, as shown in Figure 4a,b, the electric field profiles extracted at 1.57 μm and 3.68 μm both demonstrate the weak electric field characteristics of an FP cavity. In detail, the secondary and basic mode of FP resonance dominate these two resonant absorption peaks respectively (please refer to Figure A1 for more information). The first two resonant absorption peaks are thus achieved by utilizing vertical FP resonance, thereby avoiding the undesired complexity in designing the nanoresonator structures of the top Cr metasurface layer. In contrast, for the MWIR range, Figure 4c shows the electric field mainly confined beneath the largest Cr patch and along the edge of the Cr grid. Obviously, a similar electric field characteristic as seen in Figure 2d can be identified, i.e., an MD resonance mode. While the resonance mechanism seems a bit complex: the grids can intrinsically induce both ED and MD resonance, jointly contributing to the absorption at λ_3_ = 4.64 μm [36]. Overall, the combined resonance effects of EDs and MDs generated by Cr patches and grids coordinately produce the absorption peak at 4.64 μm (see Figure A2 for more information).

Finally, in the LWIR range, the electric field distributions are more straightforwardly evident. As shown in Figure 4d–g, the electric fields are intensely localized in a prominent manner centered at different patches. Moreover, the electric field profiles of the four absorption peaks in the LWIR region are respectively concentrated around four patches of different sizes, in perfect agreement with the YOZ profiles in Figure 2c. Herein, the resonant wavelength is clearly linearly proportional to the patch size. Four successively increasing resonant wavelengths in the LWIR range correspond to the four Cr patches, with sizes that increase sequentially. As indicated by the analyses in Section 2, these four LWIR resonant modes are induced by the ED resonance of patches closely coupled with each other to generate the expanded broadband absorption.

**Figure 5:**
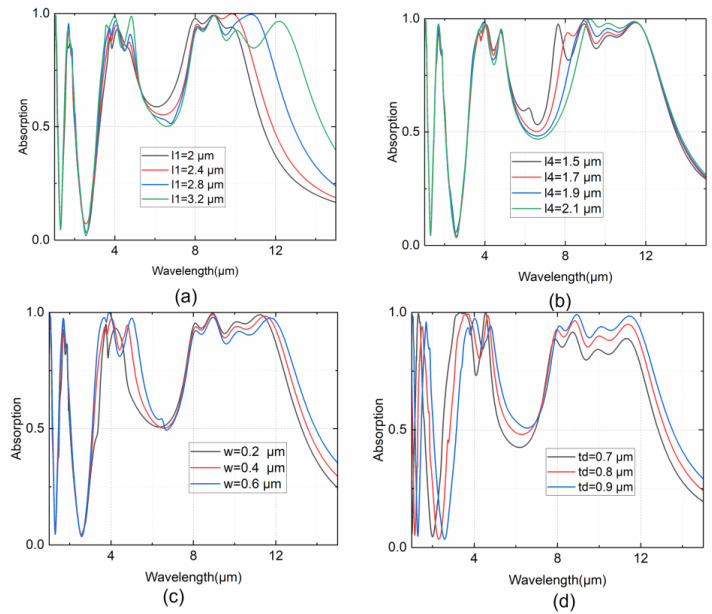
The absorption spectra extracted under a parameter sweep with varying (**a**) patch length *l*_1_, (**b**) patch length *l*_4_, (**c**) grid width *w* and (**d**) CaF_2_ thickness *t_d_*.

Based on the absorption mechanisms mentioned above, the scaling impact of the geometric dimensions to the absorption performance of our MSA is also investigated. As analyzed before, the resonance wavelength is in direct proportion to the size of the multipoles. And the thickness of dielectric layer directly determines the condition of phase matching of reflected waves for the FP mode. As shown in Figure 5a,b, the variation in the patch lengths only leads to the corresponding resonant wavelength shift in the LWIR range. For example, as shown in Figure 5b, when increasing the length *l*_4_ of the smallest patch, only the leftmost absorption peak in the LWIR region shows a significant redshift and eventually merges with the adjacent absorption peak. In contrast, other resonant peaks exhibit strong robustness against changes in the patch length, which also indicates that the four resonant peaks are independently attributed to the ED resonance of an individual Cr patch alone. Similarly, when increasing the width of the Cr grid, the increasing ED size of the grid results in a notably redshift of the second peaks in the MWIR region, as shown in Figure 5c. Notably, the redshift of λ_7_ in the LWIR region may also result from the coordinately enhanced coupling effect between the largest patch and its adjacent grids. Finally, the thickness of the CaF_2_ dielectric layer exhibits a significant impact on the absorption spectrum across the entire infrared region, as determined by the condition of impedance matching. However, only the resonant wavelengths of the first two peaks are prominently affected, indicating the dominant existence of a FP cavity mode. Spectral split is also observable in the first absorption peak of the MWIR region, which might be caused by the weak MD resonance of small patches.

**Figure 6:**
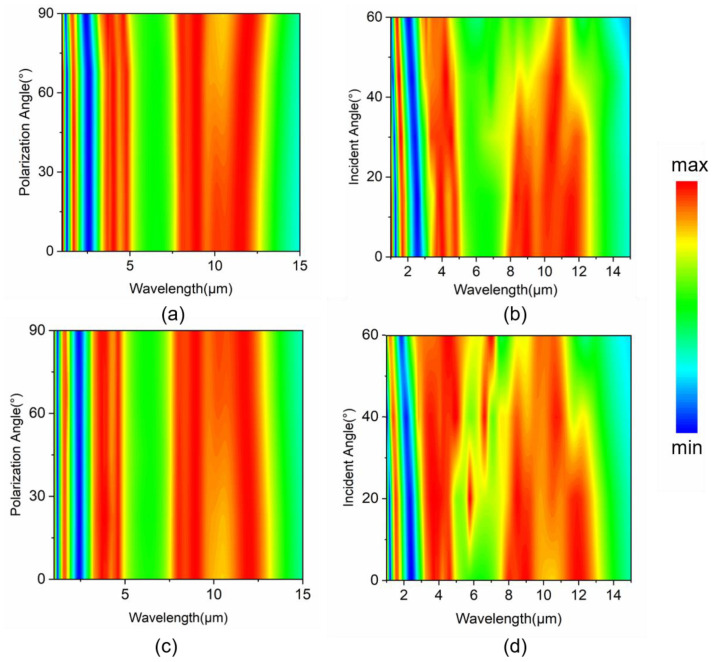
The absorption color maps obtained by different (**a**) polarization angles and (**b**) incident angles of TE incidence and (**c**) polarization angles and (**d**) incident angles of TM incidence.

Practical applications usually require insensitivity or robustness to variations in the incident angle and polarization angle of incident radiations. Here, we also probe the absorption spectra by varying the incident and polarization angles. As shown in Figure 6a,c, despite the structure of our MSA not being omni-directionally symmetric or isotropic, our scheme still exhibits excellent polarization angle insensitivity in both the TE and TM polarization mode. Across the polarization angle range from 0 to 90 degrees, the broadband absorption spectrum remains nearly unchanged. Regarding the insensitivity to incident angle variations, in TE mode, as shown in Figure 6b, the power absorption is maintained at above 80% when the incident angle is varied within 60 degrees in both the SWIR and the MWIR regions and within 45 degrees in the LWIR region. In the TM mode, as shown in Figure 6d, the high absorption in the SWIR region still holds at around 50 degrees, and the absorption bandwidth in the MWIR region even increases with a larger incident angle. In the LWIR region, the device possesses good absorption and large bandwidth at an incident angle varied within 40 degrees. It is notable that other resonance modes are seen because of the z-component electric field (6–7 μm), which does not affect the overall absorption effect in atmospheric windows. Overall, our scheme demonstrates high robustness against angle variations in the incident radiation, along with its promising performance in multi-band broadband absorption for potential applications.

## 4. Conclusions

In summary, we have proposed a scheme to construct a multi-band metasurface absorber with broadband performance for three infrared atmospheric windows. By deliberately choosing the geometric dimensions and materials of nanoresonators, we have successfully combined and coupled seven independent and low-Q resonance modes for multi-band broadband absorption control for multiple atmospheric windows. As a result, our scheme achieves multiband and broadband high absorption in the SWIR region (1.4–1.7 μm), the MWIR region (3.2–5 μm) and the LWIR region (8–13 μm) respectively, with an average absorption of approximately 90%. The electric field profiles in each resonance peak well confirm the characteristics of the second-order FP mode ($\lambda_{1}$, SWIR), the basic FP mode ($\lambda_{2}$, MWIR), the hybrid mode of ED and MD resonance ($\lambda_{3}$, MWIR) and four ED resonances with distinctly different wavelengths ($\lambda_{3}-\lambda_{7}$, LWIR) as defined. In such a manner, each absorption peak can be independently modulated by individually adjusting the corresponding geometric parameters due to independent resonance modes, ensuring the scalability and versatility of our scheme for potential applications. Finally, numerical results also confirm the robustness of our scheme in the face of variations in the polarization angle and incident angle. Such merits explicitly indicate the adaptability of our scheme for practical deployment in myriads of potential applications, including infrared imaging, photodetection, communication and so on.

## Figures and Tables

**Table 1:** Infrared metasurface absorber.

Ref.	Material	Operating Wavelength (μm)/Absorption		
		SWIR	MWIR	LWIR
[29]	Gold	-	4.28/98.2%	8.23/99.5%
[45]	Aluminum	-	3.5–4.1/>90%	-
[22]	Gold	-	-	7.8–11.9/84.7%
[46]	Gold/Si_3_N_4_	-	-	8–14/~80%
[3]	Silicon	-	-	8–14/90.36%
[47]	Silicon/Silica	1.54–1.57/~70%	-	-
[37]	ITO	4–16/>80%		
[48]	Titanium nitride	0.25–20/>90%		
[34]	Silica/Germanium	-	4–6.3/>80%	8.7–9.6/>80%
[27]	Titanium	-	3.3–4.4/52%	8.5–12.5/86%
This work	Chromium	1.4–1.7/87.6%	3.2–5/92.7%	8–13/92.4%

## Data Availability

The data presented in this study are available on request from the corresponding author.

## Appendix A

When adjusting the length of the patch in Figure A1a, two absorption peaks in the SWIR and MWIR regions are hardly affected. When l = 3.6 μm is equal to the inner diameter of the grid, there are no longer any nanoresonators existing in the top Cr layer, which means that the remaining two absorption peaks can only generated from the FP cavity. Furthermore, the phase change in the Cr layer is negligible due to its extremely small thickness compared with the wavelength. The wavelength in the basic mode (3 μm) is nearly twice as much as in the second mode (1.6 μm).

## Appendix B

As we can see from Figure A2, both a grid (blue line) and patches can produce absorption peaks in the MWIR region due to the FP and multipole resonances but with a quite limited absorption rate and bandwidth, in particular. However, when the grid and patches work together, the overall absorption efficiency is significantly improved.
